# Evaluating early response of cervical cancer under concurrent chemo-radiotherapy by intravoxel incoherent motion MR imaging

**DOI:** 10.1186/s12885-016-2116-5

**Published:** 2016-02-10

**Authors:** Li Zhu, Lijing Zhu, Hua Shi, Huanhuan Wang, Jing Yan, Baorui Liu, Weibo Chen, Jian He, Zhengyang Zhou, Xiaofeng Yang, Tian Liu

**Affiliations:** Department of Radiology, Nanjing Drum Tower Hospital, the Affiliated Hospital of Nanjing University Medical School, Nanjing, 210008 China; The Comprehensive Cancer Centre of Drum Tower Hospital, the Affiliated Hospital of Nanjing University Medical School, Nanjing, 210008 China; Philips Healthcare, Shanghai, China; Department of Radiation Oncology and Winship Cancer Institute, Emory University, Atlanta, GA 30322 USA

**Keywords:** Intravoxel incoherent motion MR imaging, Tumor response, Magnetic resonance imaging, Cervical cancer, Concurrent chemo-radiotherapy

## Abstract

**Background:**

Intravoxel incoherent motion (IVIM) MR imaging has been applied in researches of various diseases, however its potential in cervical cancer patients has not been fully explored. The purpose of this study was to investigate the feasibility of IVIM MR imaging to monitor early treatment response in patients receiving concurrent chemo-radiotherapy (CCRT) for advanced cervical cancers.

**Methods:**

Twenty-one patients receiving CCRT for advanced cervical cancer were prospectively enrolled. MR examinations including IVIM imaging (with 14 *b* values, 0 ~ 1000 s/mm^2^) were performed at 4 time points: 1-week prior to, 2-week and 4-week during, as well as immediately post CCRT (within 1 week). The apparent diffusion coefficient (ADC) maps were derived from the mono-exponential model, while the diffusion coefficient (D), perfusion fraction (*f*) and pseudo-diffusion coefficient (D*) maps were calculated from the bi-exponential model. Dynamic changes of ADC, D, *f* and D* in cervical cancers were investigated as early surrogate markers for treatment response.

**Results:**

ADC and D values increased throughout the CCRT course. Both *f* and D* increased in the first 2 to 3 weeks of CCRT and started to decrease around 4 weeks of CCRT. Significant increase of *f* value was observed from prior to CCRT (*f*_1_ = 0.12 ± 0.52) to two-week during CCRT (*f*_2_ = 0.20 ± 0.90, *p* = 0.002).

**Conclusions:**

IVIM MR imaging has the potential in monitoring early tumor response induced by CCRT in patients with cervical cancers.

## Background

Cervical cancer is the third most common malignancy in women worldwide, accounting for 9 % of the total new female cancer cases [1]. Primary treatment selection is guided by tumor stage [2]. For those who are diagnosed at the locally advanced stage, concurrent chemo-radiotherapy (CCRT) is currently the standard care [3, 4]. Because ineffective treatment is associated with increased toxicity and morbidity, accelerated tumor growth, a delay in commencing alternative, potentially effective treatment, and unnecessary expense [5], the ability to rapidly and accurately predict the response of a tumor to therapy would have immense value in clinical practice. Therefore, studies of reliable early surrogate markers of tumor response to these cancer therapies are warranted.

The past decade has witnessed rapid developments in magnetic resonance imaging (MRI). A number of studies have established MRI as the most effective imaging modality for the diagnosis of the cervical cancers as well as the assessment of tumor response to therapy [6, 7]. Conventional T1-weighted and T2-weighted MRIs offer anatomical information such as tumor size, while newer sequences such as perfusion-weighted (PW), diffusion-weighted (DW) MRIs and MR spectroscopy (MRS) have demonstrated potential as early predictors by offering a combination of morphological, physiological and metabolic information. DW-MRI allows creation of image contrast based on thermally driven motion of water molecules. It is well-established that apparent diffusion coefficient (ADC) values of malignant tumors are commonly lower than those of normal tissues or benign lesions. In addition, effective anti-cancer treatment can be reflected by increased ADC values due to alterations of tumor cellularity and integrity of cell membrane caused by necrosis and apoptosis [7–10]. A prospective study on cervical cancer showed that the post treatment ADC values of the complete response (CR) increased rapidly compared with the partial response (PR) and stable disease (SD) groups [11]. This study further demonstrated that tumor with low pretreatment ADC values tended to respond better to CCRT than those with high baseline ADC values. A similar finding was reported in a rectal cancer study by Hein et al. [12]. These studies have indicated DWI’s potential for predicting and monitoring treatment response. However, in vivo microscopic motion of water molecules is influenced by not only diffusion of water molecules, but also microcirculation of blood in the capillary network [13]. As a result, ADC values represent a combined signal of microscopic perfusion and diffusion instead of the pure diffusion.

PW-MRI provides information regarding the perfusion and permeability of tumors with time-dependent intravenous delivery of exogenous contrast agent. Dynamic contrast-enhanced MRI (DCE-MRI) has been shown useful in tumor detection as cervical tumors typically enhance intensely and early (30s) after gadolinium injection [14]. Using DCE-MRI to assess tumor response has also been reported in numerous studies [15–18], and tumors with high perfusion before therapy or increased signal intensity within the first 2 weeks after treatment appeared to indicate favorable prognosis [19]. However, the performance of DCE-MRI is more complex than other imaging techniques because the dependence of intravascular contrast media use. The unavoidable measurement error, the complicated analysis and presentation of imaging data, and potential development of nephrogenic systemic fibrosis associated with gadolinium-containing contrast material use also limited the clinical use of DCE-MRI [20].

Intravoxel incoherent motion (IVIM), initially described by Le Bihan et al. [21], was proposed as an extension of DW-MRI by using an increased number of *b *values [22]. At low *b* values, data obtained are dominated by perfusion effects, while signal delay captured at high *b* values is mainly attributed to diffusion [23]. All these features make separate analysis of pure diffusion coefficient (D) and perfusion-related incoherent microcirculation (D*) possible. In recent years, with the development in MR hardware, a renewed interest in IVIM has been shown on various organs such as head and neck [24], prostate [25], breast [26], and kidney [27]. In these studies, IVIM offered information on both tissue characterization and tumor response.

Tumor differentiation based on the IVIM model has also been demonstrated in cervical cancer with low perfusion and diffusion characteristics [28], and IVIM parameters could discriminate cervical cancer from benign tissue. However, applications of IVIM on monitoring tumor response of cervical cancer have not been reported yet. The main purpose of this study is to evaluate the feasibility of IVIM for predicting the therapeutic efficacy of treatments in cervical cancer and to investigate IVIM as an early imaging biomarker for treatment response.

## Methods

### Patient and treatment characteristics

This prospective study was approved by the Committee on Medical Ethics of Nanjing Drum Tower Hospital, and all patients enrolled signed the informed consent forms. The study inclusion criteria were: 1) women with advanced cervical cancers (i.e., clinically staged IIB to IVA based on the International Federation of Gynecology and Obstetrics (FIGO) classification) diagnosed with biopsies, 2) age older than 18 years, and 3) no prior history of cervical cancer treatment. The study exclusion criteria were: 1) patients ineligible for CCRT, such as those with pregnancy, renal or liver failure, current infection and certain drug allergies and 2) patients with MRI contraindication such as pacemaker, metal implantation and claustrophobia disorder.

All patients underwent CCRT with external beam radiotherapy (EBRT) at 1.8 ~ 2.0 Gy daily to a dose of 45 ~ 50 Gy. The volume of the EBRT depended on the nodal status as determined by radiography before the therapy. Brachytherapy was used to boost with an additional 30 ~ 40 Gy to point A (corresponding to the paracervical triangle in the medial edge of the broad ligament where the uterine vessels cross the ureter). Chemotherapy consisting of weekly nedaplatin or bi-weekly nedaplatin plus paclitaxel/docetaxel was given concomitantly with EBRT. The therapy would be stopped if the leukocytes count dropped below 3000/mm^3^, or the platelet count dropped below 80,000/mm^3^, and it was resumed once the counts rose above the levels. The duration of the chemotherapy was no more than 6 weeks, and the selection of therapeutic regimen was decided individually according to baseline health condition, tumor extent, lymph node or adjacent organ involvement.

### Magnetic resonance imaging

MR examinations were performed at 4 time points: one week prior to CCRT, at the end of the 2nd week, at the end of the 4th week during CCRT, and immediately post CCRT (within 1 week). All MR examinations were performed with a 3.0-T MRI scanner (Achieva 3.0 T, Philips Healthcare, Best, the Netherlands) with a 16-channel torso phased-array body coil. A MRI scanning protocol was developed and used for all scans in this study. Patients were asked to take clyster 2 ~ 3 h before the MRI in order to reduce artifact induced by gas and feces in the rectum. The standard sequences included axial T2-weighted turbo spin-echo (TR = 4500 ms, TE = 90 ms, matrix size = 308 × 402, field of view = 30 cm × 40 cm, slice thickness = 5 mm, intersection gap = 0.5 mm, number of signal averages (NSA) = 1), sagittal T2-weighted turbo spin-echo (TR = 4500 ms, TE = 90 ms, matrix size = 212 × 209, field of view = 30 cm × 40 cm, slice thickness = 5 mm, intersection gap = 0.5 mm, NSA =1), axialT2-weighted spectral presaturation attenuated inversion recovery (SPAIR) (TR = 4700 ms, TE = 70 ms, matrix size = 376 × 389, field of view = 20 × 20 cm, slice thickness = 5 mm, intersection gap = 0.5 mm, NSA = 1), sagittal T2-weighted SPAIR(TR = 4700 ms, TE = 70 ms, matrix size = 256 × 179, field of view = 20 × 20 cm, slice thickness = 5 mm, intersection gap = 0.5 mm, NSA = 1), 3D T1-weighted turbo-field-echo contrast-enhanced acquisition (TR = 3.0 ms, TE = 1.42 ms, field of view = 256 × 194 mm, matrix size = 30 cm × 40 cm, slice thickness = 1.5 mm, intersection gap = 0 mm, NSA = 1). Intravenous bolus injection of 0.1 ~ 0.2 mmol/kg body weight gadodiamide was performed at a rate of 3.0 ml/s, followed by a 15 ml saline flush with high pressure injector after contraindications such as severe renal failure and liver transplantation had been excluded. The scanning time of IVIM was approximately 10 min and the total scanning time was about 30 min.

All the examinations were acquired with free breathing. Fourteen *b* values (0, 10, 20, 30, 40, 50, 100, 150, 200, 350, 500, 650, 800, 1000 s /mm^2^) were used in the axial single shot diffusion weighted echo planar imaging (SS-EPI) (TR = 2834 ms,TE = 105 ms, matrix size = 152 × 120, field of view = 30 × 40 cm, slice thickness = 6 mm, intersection gap = 0.5 mm, NSA = 1).

### Image and data analysis

All MR images were independently analyzed by 2 experienced radiologists (Jian He, Zhengyang Zhou) with 6 and 8 years’ experience in gynecology. The radiologists were blinded to each other’s reading. The dataset was analyzed based on the bi-exponential IVIM model introduced by Le Bihan [29] with the following function: *S*_*b*_/*S*_0_ = (1 – *f*) * exp(-b * D) + *f* * exp(-*b* * (D* + D)), in which *S*_*b*_ represents the mean signal intensity with diffusion gradient *b*, *S*_0_ represents the mean signal intensity when *b* = 0 s/mm2. The IVIM data was evaluated using DWI-Tool developed by Philips with IDL 6.3 (ITT Visual Information Solutions, Boulder, CO) for D, *f* and D* maps. The tumor on MRI was defined as a mass with higher signal intensity than the adjacent cervical stroma yet lower signal intensity than the fluid signal in the urinary bladder on a T2-weighted image [30]. The specific slice of DWI with the biggest tumor section was selected referring to the corresponding axial T2-weighted images, and then, a region of interest (ROI) was manually drawn as large as possible along the inside of the tumor margin. The macroscopic necrotic areas, large vessels and areas with artifacts induced by air-water interface were excluded during the tumor contour. The longest diameter of the tumor was subsequently measured. If no residual tumor was observed after treatment, five equal-sized ROIs (each 5 mm^2^) were placed within the solid components of the tumor region prior to treatment, and the diameter of the lesion would be recorded as 0 cm. The ADC values were derived from the mono-exponential model, while the D, *f* and D* values were calculated with bi-exponential model. The mean values of the two radiologists’ measurement were calculated as the final results. The ROIs were transferred to the corresponding IVIM parametric maps with the Image J (NIH, Bethesda, MD, USA).

### Treatment outcome analysis

According to the evaluation criteria in solid tumors (RECSIT) [31], response to treatment was decided by the shrinkage of tumor size. Tumor response was classified into four groups: (1) complete response (CR) was concluded if no residual tumor can been seen on the MRI images; (2) partial response (PR) was concluded if an over 30 % size reduction of the tumor was observed as compared with the original size; (3) progress disease (PD) was concluded if there was at least 20 % increase in the longest diameter of tumor in comparison with the pre-treatment size; (4) stable disease (SD) was concluded if there was neither sufficient decrease to qualify for PR nor sufficient increase to qualify for PD.

### Statistical analysis

All the statistical analysis was performed using SPSS 16.0 (SPSS Inc., Chicago, IL). Significant changes of IVIM parameters with time were tested using paired *t* test. In order to evaluate the correlations between IVIM parameters, the Pearson’s correlation coefficient was calculated with 95 % confidence interval. Two-tailed *p* values were used and *p* values less than 0.05 were considered as statistically significant. An intraclass correlation coefficient (ICC) was calculated to evaluate inter-observer reliability.

## Results

From December 2013 to January 2015, 21 patients with locally advanced cervical cancers were enrolled in this prospective study, and all of them were confirmed histologically as squamous cell carcinoma. The patient and treatment characteristics are summarized in Table 1. Eighteen of those patients were classified as CR and three as PR after the treatment. Clinical information at four time points was collected from all patients.

**Table 1:** Patient and treatment characteristics

Clinical features	Values
No of patients	21
Age (years)	49.6 (24–76)
FIGO stage:	
II	11 (52.4 %)
III	6 (28.6 %)
IV	4 (19.0 %)
Metastasis (*n* = 4)	
Bladder	1 (4.8 %)
Rectum	3 (14.3 %)
Treatment outcome	
Complete response	18 (85.7 %)
Partial response	3 (14.3 %)

Data are N (%) or mean (range)

*FIGO* the International Federation of Gynecology and Obstetrics

**Fig. 1:**
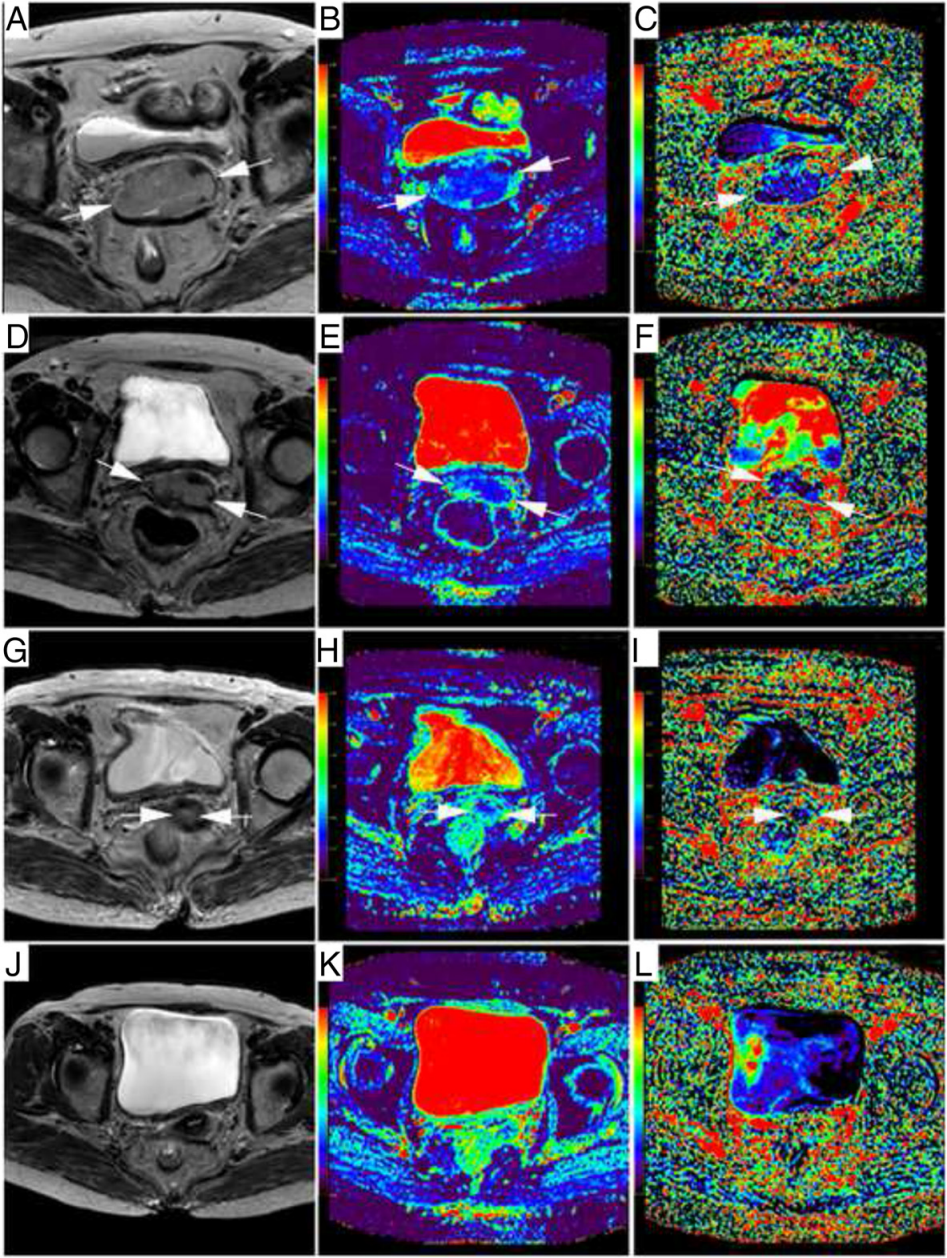
MR images of a patient receiving concurrent chemo-radiotherapy (CCRT) for advanced cervical cancer (FIGO stage IIIA). **a** Axial T2-weighted image before CCRT shows a hyperintense mass lesion (arrows) at the cervix, with a maximal diameter of 6.0 cm. **b** ADC map corresponding to (**a**) shows a low ADC value (0.933 × 10^−3^ mm^2^/s) of the lesion compared with the adjacent normal cervical tissues (1.54 × 10^−3^ mm^2^/s). **c** The *f* map corresponding to (**a**) shows a low *f* value (0.097) of the lesion compared with the adjacent normal cervical tissues (0.198); **d** Two weeks after initiation of CCRT, the diameter of cervical cancer decreased to 4.2 cm. **e** The ADC value of the lesion corresponding to (**d**) increased to 1.038 × 10^−3^ mm^2^/s compared with (**b**). **f** The *f* value of the lesion corresponding to (**d**) increased to 0.116 compared with (**c**). **g** One month after initiation of CCRT, the lesion continues to decrease with a diameter of 1.7 cm. **h** The ADC value of the lesion corresponding to (**g**) continues to increase to 1.563 × 10^−3^ mm^2^/s compared to (**e**). **i** The *f* value of cervical cancer corresponding to (**g**) continues to increase to 0.239 compared to (**f**). **j** Post CCRT, no residual lesion is observed in the axial T2-weighted image. **k** The ADC value of the lesion corresponding to (**j**) continues to increase to 1.737 × 10^−3^ mm^2^/s compared to (**h**). **l** The *f* value of cervical cancer corresponding to (**g**) continues to increase to 0.165 compared to (**i**)

The mean values of diameter, D, *f*, D*, and ADC obtained for all patients over time are shown in Table 2. The initial mean tumor size was 4.17 ± 1.23 cm, and the diameter decreased significantly after treatment. All 21 patients enrolled achieved efficient local control at the end of therapy, with no one classified as PD or SD. Temporal reduction of lesions is shown on the IVIM parameter maps (Fig. 1).

**Table 2:** Changes of IVIM parameters and tumor size during the concurrent chemo- radiotherapy course

Variables	Time point 1	Time point 2	Time point 3	Time point 4
Diameter (cm)	4.17 ± 1.23	2.56 ± 1.26	1.14 ± 1.12	0.20 ± 0.53
*f*	0.12 ± 0.52	0.20 ± 0.90	0.22 ± 0.79	0.18 ± 0.58
D* (×10^−3^ mm^2^/s)	29.23 ± 26.49	33.73 ± 21.76	34.80 ± 21.00	34.44 ± 17.30
ADC (×10^−3^ mm^2^/s)	1.00 ± 0.11	1.39 ± 0.26	1.66 ± 0.17	1.67 ± 0.16
D (×10^−3^ mm^2^/s)	0.85 ± 0.12	1.09 ± 0.14	1.31 ± 0.11	1.41 ± 0.13

*ADC* apparent diffusion coefficient, *D* diffusion coefficient, *f* perfusion fraction, D* pseudo-diffusion coefficient

**Fig. 2:**
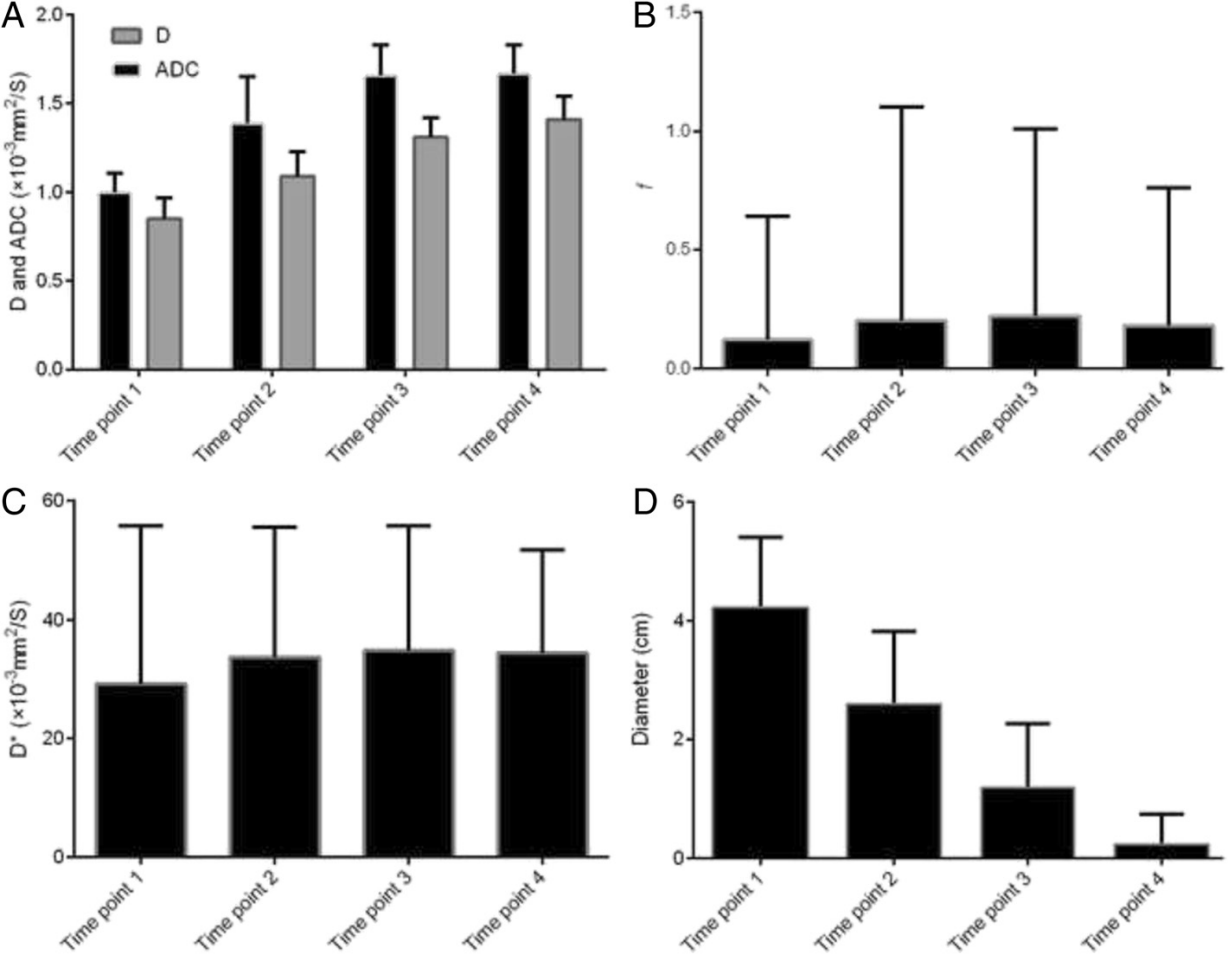
Dynamic changes of various MR parameters of cervical cancers during concurrent chemo-radiotherapy (CCRT). **a** D and apparent diffusion coefficient (ADC) values of cervical cancers increase during the course of CCRT and the D values are lower than the ADC values. **b** *f* values of cervical cancers increases initially and decreases after 4 weeks of therapy. **c** D* values of cervical cancers share the same tendency as *f*. **d** Tumor sizes shrink over the course of CCRT. Time point 1, before CCRT within one week; time point 2, at the end of the second week of CCRT; time point 3, at the end of the first month of CCRT; time point 4, immediately after CCRT within one week

All IVIM parameters showed big increases at week 2 during CCRT, values of D and ADC kept increasing during CCRT, while values of *f* and D* went down after week 4 during CCRT (Fig. 2). A positive correlation between D and ADC values was demonstrated at each time point (*p*_1_ < 0.001, *p*_2_ = 0.003, *p*_3_ = 0.032, *p*_4_ < 0.001 respectively), but no significant correlations between other parameters were found in this study. While comparing the values at different time points, no significant difference was found between the ADC values at time points 3 and 4 (*p* = 0.879), *f* values at time points 2 and 3, as well as 2 and 4 (*p*_23_ = 0.408, *p*_24_ = 0.337), and among D* values of all time points (*p*_12_ = 0.557, *p*_13_ = 0.461, *p*_14_ = 0.480, *p*_23_ = 0.875, *p*_24_ = 0.913, *p*_34_ = 0.954). But all the parameters showed remarkable statistical differences between the rest time points (all *p* < 0.05) indicating detectable changes in IVIM parameters during CCRT. The detailed results were shown in Table 3.

**Table 3:** Differences of IVIM parameters between different time points in locally advanced cervical cancer patients under concurrent chemo- radiotherapy (CCRT)

Variables	Time point 1 versus 2	Time point 1 versus 3	Time point 1 versus 4	Time point 2 versus 3	Time point 2 versus 4	Time point 3 versus 4
Diameter	<0.001	< 0.001	< 0.001	< 0.001	< 0.001	< 0.001
D	< 0.001	< 0.001	< 0.001	< 0.001	< 0.001	0.016
ADC	< 0.001	< 0.001	< 0.001	< 0.001	< 0.001	0.879
*f*	0.002	< 0.001	0.005	0.408	0.337	0.047
D*	0.557	0.461	0.480	0.875	0.913	0.954

Data are shown as *p* value from each comparison. Time point 1, before CCRT within one week; time point 2, at the end of the second week of CCRT; time point 3, at the end of the first month of CCRT; time point 4, immediately after CCRT within one week

*ADC* apparent diffusion coefficient, *D* diffusion coefficient, *f* perfusion fraction, D* pseudo-diffusion coefficient

The ICC between two observers was 0.964 (95 % CI, 0.887 ~ 0.988; *p* < 0.001), and the ROI area was 895.82 ± 596.51 mm^2^ (range: 160.20 ~ 2037.45 mm^2^)

## Discussion

In this study, we demonstrated the potential of IVIM MR imaging in monitoring early CCRT response of cervical cancer. To the best of our knowledge, this is the first report on such an application in cervical cancer.

In cervical cancer, DWI was first reported as a diagnostic tool to distinguish the malignancy from normal uterine cervix [32]. In our study, the initial ADC and D values of cervical cancer (ADC = 1.00 ± 0.11 × 10^−3^mm^2^/s, D = 0.85 ± 0.12 × 10^−3^mm^2^/s) were in line with the published results (ADC = 0.99 ± 0.18 × 10^−3^mm^2^/s, D = 0.86 ± 0.16 × 10^−3^mm^2^/s) [28] which were significantly different from normal cervical tissues (ADC = 1.57 ± 0.17 × 10^−3^mm^2^/s, D = 1.32 ± 0.12 × 10^−3^mm^2^/s). The low ADC and D values of cervical cancer were due to the increased cellularity, which was an important factor that influences the extracellular space and microscopic water diffusion in tumor tissue. In tumor tissues, the ADC values were higher than D values, which suggested that a mono-exponential model could overestimate the water diffusion in the cancerous tissue, because of the “contamination” of ADC as a combined signal measurement of the microscopic perfusion and diffusion.

Previous studies [8, 33] have demonstrated that effective anticancer treatment would result in an increased water diffusion. Findings in this study supported the association between the ADC change and post-therapy response. Similarly, we demonstrated that the mean ADC of responders increased after treatment. D value changed with ADC value and both increased over the course of treatment. Significant differences in ADC values were demonstrated at various time points except for the last stage of therapy (*p* = 0.879). Less restricted motion of water molecules induced by damages in tumor tissue (e.g., loss of cell membrane integrity and a subsequent reduction in tumor cell density) after successful treatment may explain these changes [34]. These changes appeared as early as 2 weeks during CCRT, indicating the sensitivity of ADC and D as surrogate markers of early tumor response.

In this study, the pre-treatment perfusion fraction *f* (0.12 ± 0.52) of cervical cancer was close to 0.149 ± 0.26 as reported by Lee EY et al. [28]. Significant higher initial *f* values were displayed in head-and-neck cancer patients with poor clinical outcome [24]. As no patients were classified as SD or PD in this study, a further research with a larger sample size is warranted. At follow-ups, *f* significantly increased after two-week of CCRT (*p* = 0.002) making it a potential early indicator for post-therapy response. As an imaging biomarker for the vascular compartment, the *f* value most likely reflects the vascular volume fraction of the tumor instead of the accurate blood volume/plasma volume, it is mainly related to the regional blood flow in DCE-MRI. Also by decreasing cell density and modulating the tumor microenvironment, CCRT is considered to improve the blood supply and oxygenation status of tumor cells, resulting in a substantial reduction of radiation-resistant hypoxic tumor cells [35]. Therefore the tumor perfusion is associated with the effect of CCRT. Although Shibuya K et al [36] have shown that blood flow increased after 20 Gy of radiation therapy in cervical cancer, Ganten M K et al [37] and Gaeta M et al [38] found no significant changes of *f* over time in rectal cancers. This contradiction may be explained by different histopathological features and capillary network distribution in different types of tumors. Moreover, such discrepancy could also be caused by different imaging techniques and treatment regimes in the clinical studies.

During CCRT, the *f* initially increased and later decreased around 4 weeks into therapy. This decline may be explained by the hypothesis that: local fibrosis and angiolysis (vessel occlusion, reduction, or disappearance) consequent on the large cumulative radiation dose altered the microcirculation in various ways, and replaced the leading role of cell lysis in the early period, and the perfusion changes comprise a greater percentage as a result. Previous study in lung has shown the role of various early inflammatory proteins in stimulating proliferation and migration myofibroblasts during later fibrosis [39]. As most patients finished their EBRT at the 5th week, transformation of therapy regimen may be answerable to the alteration between time point 3 and 4, whereas further histopathological examinations are needed for verification.

In tumors, D* reflects the rate of microcapillary blood flow and is positively related to f according to the double exponential model theory. Our study supported this observation by showing a changing D* in agreement with *f*. As D* value is well known for its huge standard deviation, data instability and its dependence on signal-to-noise ratio (SNR), wider clinical application is limited in this situation. A recent IVIM imaging study of hepatocellular carcinoma has shown poor reproducibility of D* with a mean coefficient of variation equal to 60.6 % [40]. Once further improvement is achieved to the robustness and reproducibility of D*, it might be suitable to bring it into estimating the tumor response.

There are several limitations in this study. First is the relatively small sample size of patients, only 21 patients were involved in this prospective study. What’s more, all patients enrolled were histologically confirmed as squamous cell carcinoma without adenocarcinoma or other types of carcinoma. Most participants in our study were staged as II (52.4 %), patients clinically classified as III or IV is relatively few. All these factors made our sample lack of representative, a source where bias may occur. Nevertheless, the preliminary results were promising and further studies with a larger and more diverse cohort are warranted. A second limitation of this study was that the appropriate number of *b* values suitable for cervix is still unknown. Various numbers were used in the previous studies and we used 14 *b* values in this study. So searching for the best selection of *b* values in cervix imaging would be another optimization strategy in further studies. Thirdly, without histological confirmation MRI could be inaccurate at times. For instance, a residual tumor and post-treatment fibrosis may be difficult to differentiate. Since multiple biopsies are impractical, future animal experiments could help us better understand early tumor response.

## Conclusions

IVIM MR imaging has shown dynamic changes of cervical cancers during treatment, which makes IVIM parameters as potential biomarkers for tumor response following cervical cancer CCRT. Clinical studies with a large cohort to confirm these promising results are warranted. With technological advances, IVIM will become a valuable imaging tool in the clinic as well as in cancer research.

## Abbreviations

ADC: apparent diffusion coefficient
CCRT: concurrent chemo-radiotherapy
CR: complete response
D: diffusion coefficient
D*: pseudo-diffusion coefficient
DCE: dynamic contrast-enhanced
DW: diffusion-weighted
EBRT: external beam radiotherapy
*f*: perfusion fraction
FIGO: international federation of gynecology and obstetrics
IVIM: intravoxel incoherent motion
MRI: magnetic resonance imaging
NSA: number of signal averages
PD: progress disease
PR: partial response
ROC: receiver operating characteristics
ROI: region of interest
SD: stable disease
SD: standard deviation
SNR: signal-to-noise ratio
SPAIR: spectral presaturation attenuated inversion recovery
SS-EPI: single shot diffusion weighted echo planar imaging
